# Control of Bone Resorption by Semaphorin 4D Is Dependent on Ovarian Function

**DOI:** 10.1371/journal.pone.0026627

**Published:** 2011-10-26

**Authors:** Romain Dacquin, Chantal Domenget, Atsushi Kumanogoh, Hitoshi Kikutani, Pierre Jurdic, Irma Machuca-Gayet

**Affiliations:** 1 Institut de Génomique Fonctionnelle de Lyon, Université de Lyon, CNRS, Ecole Normale Supérieure de Lyon, Lyon, France; 2 Department of Immunopathology, Research Institute for Microbial Diseases, Osaka University, Suita, Osaka, Japan; Laboratoire Arago, France

## Abstract

Osteoporosis is one of the most common bone pathologies, which are characterized by a decrease in bone mass. It is well established that bone mass, which results from a balanced bone formation and bone resorption, is regulated by many hormonal, environmental and genetic factors. Here we report that the immune semaphorin 4D (*Sema4D*) is a novel factor controlling bone resorption. *Sema4D*-deficient primary osteoclasts showed impaired spreading, adhesion, migration and resorption due to altered ß3 integrin sub-unit downstream signaling. In apparent accordance with these *in vitro* results, *Sema4D* deletion in sexually mature female mice led to a high bone mass phenotype due to defective bone resorption by osteoclasts. Mutant males, however, displayed normal bone mass and the female osteopetrotic phenotype was only detected at the onset of sexual maturity, indicating that, *in vivo*, this intrinsic osteoclast defect might be overcome in these mice. Using bone marrow cross transplantation, we confirmed that *Sema4D* controls bone resorption through an indirect mechanism. In addition, we show that *Sema4D* −/− mice were less fertile than their WT littermates. A decrease in Gnrh1 hypothalamic expression and a reduced number of ovarian follicles can explain this attenuated fertility. Interestingly, ovariectomy abrogated the bone resorption phenotype in *Sema4D* −/− mice, providing the evidence that the observed high bone mass phenotype is strictly dependent on ovarian function. Altogether, this study reveals that, *in vivo*, *Sema4D* is an indirect regulator of bone resorption, which acts via its effect on reproductive function.

## Introduction

Osteoporosis is one of the most common bone pathology, which is characterized by a decrease in bone mass, leading to an increased fracture risk. Therefore, slowing the osteoporosis progression has become a major health issue in the aging population of the northern hemisphere. Reproductive hormones fluctuations have a well-established role in osteoporosis. In early menopause, the acute phase of estrogens deficiency is associated with an increased bone loss [1]. However, clinical studies, have provided evidence that the etiology of this disease is more complex than just a question of estrogen withdrawal and that overall bone mass is influenced by many environmental, but also genetic factors [2]. Among those factors, several studies have suggested a role for semaphorin family members in the control of bone mass. *Sema3B* expression in osteoblasts is induced by vitamin D3 and its deficiency leads to osteopenia, secondary to increased osteoclastogenesis [3]. Sema7A is also expressed in bone cells [4]. *Sema7A* as well as *Plexin A2* (Sema 3A receptor) polymorphisms have been associated with decreased bone mineral density and increased fracture risk in postmenopausal Korean woman [5], [6]. Deficiency in *Plexin A1*, the *Sema6D* receptor, leads to increased bone mass due to a defect in osteoclast differentiation [7].

*Sema4D* is a membrane-bound class 4 semaphorin that was first identified in the immune synapse where it mediates cell-to-cell interactions and regulates the immune response [8]. *Sema4D* is in fact expressed in other tissues including platelets, ovaries and the nervous system, where it has several functions [9]. *Sema4D* stimulates both *in vitro* and *in vivo* angiogenesis [10], [11]. In the nervous system, *Sema4D* is involved in neuronal migration and neurite outgrowth. For example, *Sema4D* has been shown to regulate gonadotropin hormone-releasing hormone-1 (*Gnrh1*) positive neuron migration through the *PlexinB1-Met* complex [12], [13]. *Sema4D* acts on another level of the hypothalamic-hypophyseal-ovarian axis, as *ex vivo* data has shown that *Sema4D* is involved in mouse ovary follicle maturation [14]. Interestingly, structural studies have revealed strong similarities between the *Sema4D* homodimer and the αVß3 integrin heterodimer, which is the most abundant integrin receptor expressed in bone resorbing osteoclasts [15]. αVß3 integrin plays a major role in osteoclast function, both *in vitro* and *in vivo*. For instance, integrin ß3 −/− mice become progressively osteosclerotic with age due to dysfunctional osteoclasts that fail to polarize correctly and develop abnormal ruffled membranes [16]. In the present study, using the loss of function approach, we addressed the question whether *Sema4D* could also play a role in bone resorption.We first show that *Sema4D* is expressed in osteoclasts and that like *ß3integrin*-deficient osteoclasts, *Sema4D* −/− osteoclasts exhibit a resorption defect consecutive to reduced migration and spreading. We then provide evidence that *Sema4D−/−* females displayed a high bone mass phenotype associated to a bone resorption defect. Interestingly this high bone mass phenotype was not found in males and in sexually immature female. Besides this phenotype, we showed that *Sema4D−/−* females exhibited a reproductive defect. Finally, we demonstrate that the bone resorption phenotype does not result from an osteoclast-specific defect but that it is strictly dependent on *Sema4D* action on ovarian function. Thus *Sema4D* is an indirect regulator of bone resorption that acts through its effect on the reproductive system in females.

## Results

### *Sema4D* expressed in osteoclasts, delays osteoclasts differenciation and affects osteoclast function

**Figure 1:**
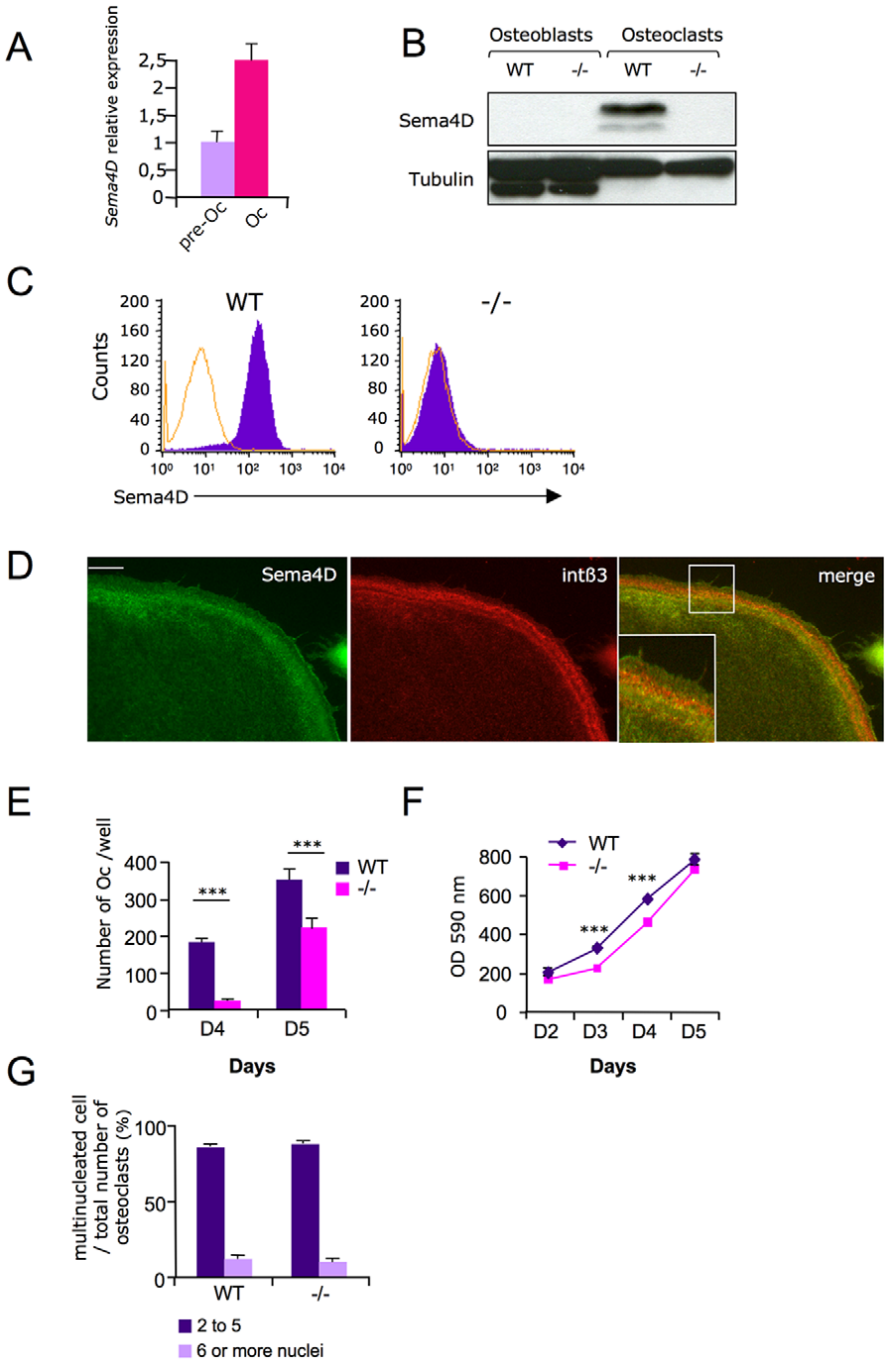
*Sema4D* is expressed in osteoclasts and its deletion delays osteoclasts progenitors proliferation and differentiation *in vitro*. A–D) *Sema4D* expression and localization. A/ QPCR analysis showing an increase of *Sema4D* expression along the osteoclast differentiation pathway, B/ Total cell extract, either from primary calvaria derived osteoblast cultures or from osteoclasts isolated from WT or *Sema4D*-deficient mice, were immunoblotted using an anti-*Sema4D* specific antibody. *Sema4D* is present in WT osteoclasts. α-tubulin was used as loading control. C/ FACS analysis on non-permeabilized pre-osteoclasts shows that *Sema4D* is expressed at the WT cell surface but not in *Sema4D* −/− cells. D/ Mature osteoclasts were fixed and immunolabeled with anti-*Sema4D* or anti-ß3 integrin chain antibodies. Merge picture and inset show that *Sema4D* partially colocalized with the ß3 integrin chain (bar: 10 µm). E–G) *Sema4D* −/− osteoclast progenitors differentiation and proliferation rate. E/ Bone marrow precursor cultured for either 4 or 5 days in the presence of MCSF and RANKL. Cultures were then stained for TRAP activity and TRAP positive multinucleated cells formed were counted. This showed that the formation of TRAP positive multinucleated osteoclasts from *Sema4D* −/− precursors was reduced compared with WT. F/ Bone marrow precursors were cultured in the presence of MCSF to assess proliferation of progenitor cells. At each time point, the cells were fixed and stained with crystal and the 590 nm OD measured, reflecting the cell number. This shows that *Sema4D* −/− precursors proliferate more slowly than their WT counterparts during the growth phase. G/ To confirm that the delay in *Sema4D* −/− osteoclast formation could be due to their impaired proliferation rate, 20% more *Sema4D* −/− bone marrow precursors (375 cells/mm^2^) were seeded than WT (312 cells/mm^2^) in the presence of M-CSF and RANKL. Mature multinucleated TRAP positive osteoclasts were counted on day 5. Similar osteoclast counts were obtained in both cultures and they displayed the same degree of multinucleation. Error bars represent SEM, *** indicate a p value≤0.001.

The most abundant integrin in osteoclasts is αVß3 integrin, which plays a major role in regulating the bone resorption process [17]. Because *Sema4D* homodimers share many structural similarities with the αVß3 integrin heterodimer [15], we hypothesized that *Sema4D* could have similar functions. We found that *Sema4D* mRNA, as well as the protein, is expressed in pre- and in mature primary osteoclasts (Figs. 1A–B) [18]. In contrast, *Sema4D* was not detected in primary osteoblasts derived from mouse calvaria (Fig. 1B). Moreover, FACS analysis showed that *Sema4D* is expressed at the osteoclast cell membrane. We further localized *Sema4D* within cytoskeletal structures by specific ß3 integrin co-immunolabeling in mature osteoclasts (Figs. 1C–D). As expected, anti-β3 integrin labeled the podosome belt at the periphery of mature osteoclasts and *Sema4D* partially colocalized with ß3 integrin. These observations were compatible with a role for *Sema4D* in osteoclast mediated bone resorption. To explore the potential effect of *Sema4D* on osteoclast differentiation, we first carried out classical *in vitro* primary osteoclast differentiation assays in the presence of M-CSF and RANKL. Osteoclast differentiation assays of WT or *Sema4D*-deficient bone marrow isolated precursors revealed that differentiation was reduced in the absence of *Sema4D*, as the number of osteoclasts measured in WT cultures was higher that in *Sema4D* −/− on days 4 and 5 (Fig. 1E). In order to assess if the reduced number of osteoclasts obtained in *Sema4D*−/−cultures was due to a proliferation or a fusion defect in progenitor cells, we first performed a proliferation test on osteoclast precursors isolated from *Sema4D* −/− or WT bone marrow in the presence of M-CSF. Crystal violet proliferation tests revealed that bone-marrow derived precursors isolated from *Sema4D*-deficient mice exhibited a small but statistically significant proliferation delay at early time points, which was abrogated after 5 days in culture (Fig. 1F). This later result strongly suggested that the fusion process had decreased, as a consequence of a slightly lower proliferation of *Sema4D* −/− precursors. To definitively prove that the reduced number of *Sema4D* osteoclasts was not due to an intrinsic fusion defect, but to proliferation, we plated 20% more *Sema4D* −/− precursors than WT in the presence of MCSF and RANKL to overcome their slower proliferation rate. Under such plating conditions, the same number of mature osteoclasts was obtained. Indeed, they contained the same number of nuclei as WT, showing that under such conditions the process of differentiation was not affected by *Sema4D* deficiency (Fig. 1G). These plating conditions allowed us to really compare *Sema4D* −/− and WT osteoclasts from the same differentiation stage, and have been used all along this study.

**Figure 2:**
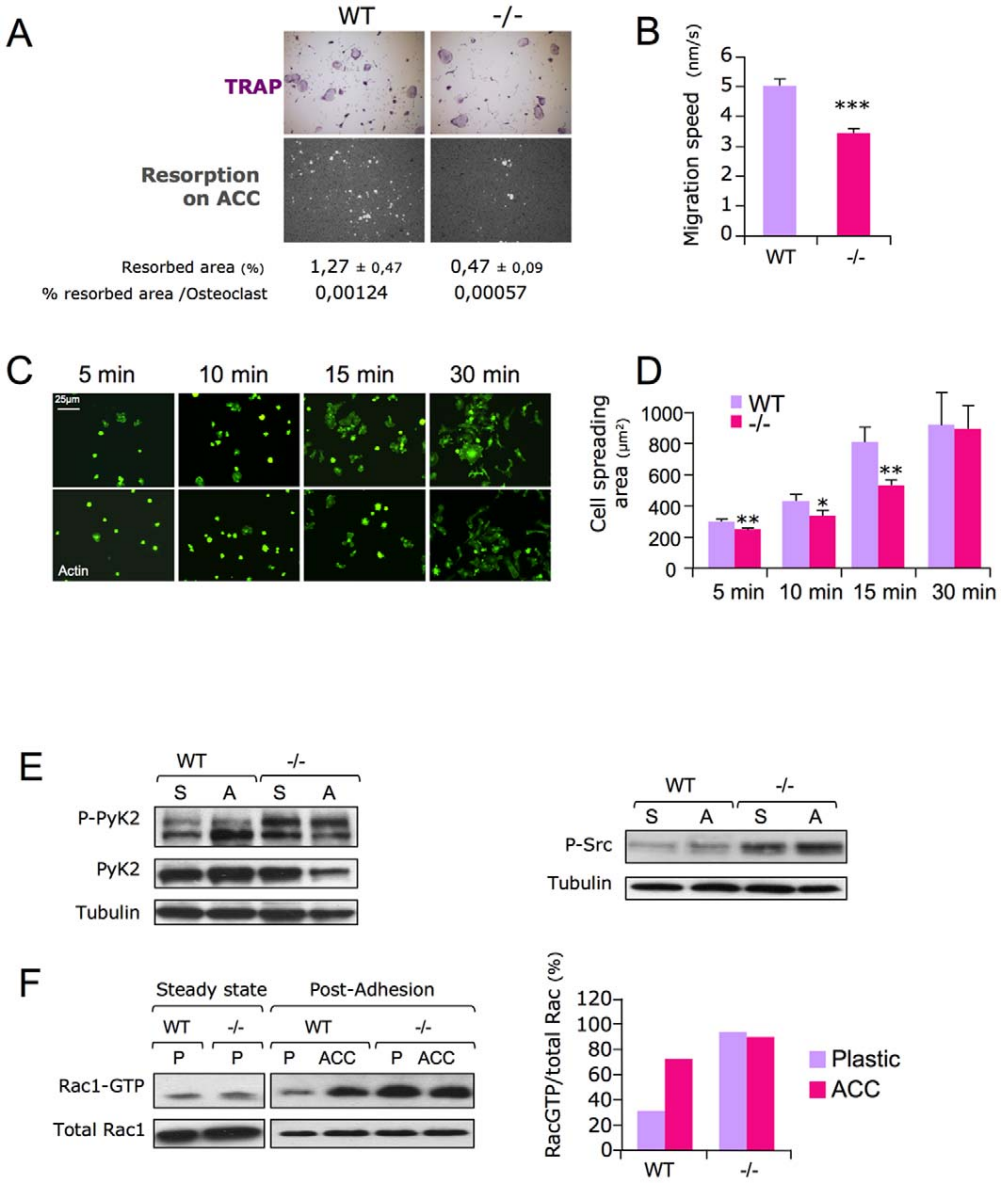
*Sema4D* −/− osteoclasts resorb less than WT and display an altered signaling of ß3 integrin sub-unit mediated adhesion. F/ A/ Resorption tests: osteoclasts were seeded on apatite collagen complex (ACC) coverslips or on plastic. 24 hours after seeding, TRAP staining was performed to assess the number of multinucleated osteoclasts (3 or more nuclei). ACC coverslips were then stained with silver nitrate to measure the percentage of resorbed area (white) versus the total area with ImageJ software micrographs are representaive of several independent experiments. B/ The migration speed of resorbing osteoclasts on ACC was measured by videomicroscopy, followed by software-assisted quantification. C/ Cell spreading ability was assessed by seeding WT and *Sema4D*-deficient osteoclasts on vitronectin- coated coverslips for the stated time. D/ The F-actin content was labeled with 488-Alexa-Phalloidin and the surface area measured using ImageJ software. Results are representative of 3 independent experiments, n≥150 (n = cell number per time point). Error bars represent SEM; * p≤0.01; ** p≤0.001; *** p≤0.0001. E/ Hyperphosphorylation of Pyk2 and c-Src in *Sema4D* −/− osteoclasts compared to WT. Osteoclasts were detached and either maintained in suspension (S) or plated on vitronectin for 30 min and lysed. The Pyk2 and c-Src phosporylation levels were revealed on total cell lysates by immunoblotting with anti-P-Pyk2 or anti-P-c-Src antibodies. This shows hyperphosphorylation of Pyk2 and c-Src in *Sema4D* −/− osteoclasts compared to WT. Increased Rac1 activity in *Sema4D* −/− pre-osteoclasts versus WT. F/ Four-hour-starved pre-osteoclasts were lifted and replated either on plastic (P) or Apatite Collagen Complex (ACC). 1 hour post-adhesion, cells extracts were pulled down for the GTP-bound Rac1 content. Rac1-GTP and total Rac1 were detected by immunoblotting and the corresponding signals were densitometrically quantitated, for steady state analysis, no treatment was applied to cells prior the GTP-Rac1 pull down. Results are representative of at least 3 independent experiments.

To then test whether osteoclast function could be affected in the absence of *Sema4D*, we performed resorption tests on an apatite collagen complex (ACC) and showed that in the absence of *Sema4D*, the percentage of resorbed area per osteoclast was significantly reduced, by two fold, in the *Sema4D*-deficient osteoclasts compared to WT (Fig. 2A). As resorption by osteoclasts is a multistep process that requires adhesion, spreading, resorption per se, and migration [19], we wanted to determine which of these steps was affected in the absence of *Sema4D*. Measurements by time-lapse video microscopy of WT and *Sema4D* −/− mature osteoclasts (n≥80) revealed that mature *Sema4D*-deficient osteoclasts migrated more slowly than WT osteoclasts (Fig. 2B). Cell spreading was delayed in the absence of *Sema4D* −/− as cell area was reduced during the early stages of adhesion on vitronectin. This difference was not observed at the latest time point, indicating that *Sema4D* −/− osteoclasts were able to make up the delay (Fig. 2C). Altogether, these data indicate that *Sema4D*-deficient osteoclasts migrate more slowly than WT and subsequently resorb mineralized matrix less efficiently than WT osteoclasts.

### Altered αVß3 integrin mediated adhesion dependent signaling in *Sema4D* −/− osteoclasts

Osteoclast migration, adhesion and resorption are highly linked processes. They are all promoted by dynamic cytoskeletal reorganization, which involves αVß3 integrin-dependent signaling. Having shown that spreading, migration and resorption are affected in *Sema4D* −/− osteoclasts, we asked whether down-stream signaling in response to αVβ3 integrin activation could normally occur in osteoclasts lacking *Sema4D*. To address this question, we analyzed c-Src and Pyk2 phosphorylation, both molecular components of the adhesion complex triggered by αVß3 integrin engagement. Osteoclasts cultured with M-CSF and RANKL for four days were detached and either maintained in suspension or layered on vitronectin coated plates for 30 min. Hyper-phosphorylation of both kinases was observed in the *Sema4D* −/− osteoclasts, either maintained in suspension or adherent as if they were constitutively active in the mutant cells (Fig. 2E). We sought to determine whether further αVβ3 down-stream signalling was also perturbed. Rac1-GTPase regulates cellular actin-dynamics, and it is the downstream target of αVß3 induced cytoskeleton reorganization in osteoclasts [20]. In accordance with c-Src and Pyk2 hyper-phosphorylation, we found that the Rac1-GTP content was higher in *Sema4D* −/− osteoclasts, regardless of the substratum (Fig. 2F), whereas the GTP-bound Rac1 level in steady-state osteoclasts was similarly low in both WT and *Sema4D* −/−. In summary, a *Sema4D* deficiency in primary osteoclasts led to reduced resorption activity, in part due to dysfunction of the adhesion properties and an unbalanced regulation of ß3 integrin sub-unit signaling.

### *Sema4D* deficiency leads to high bone mass phenotype due to decreased bone resorption in sexually mature female mice

**Figure 3:**
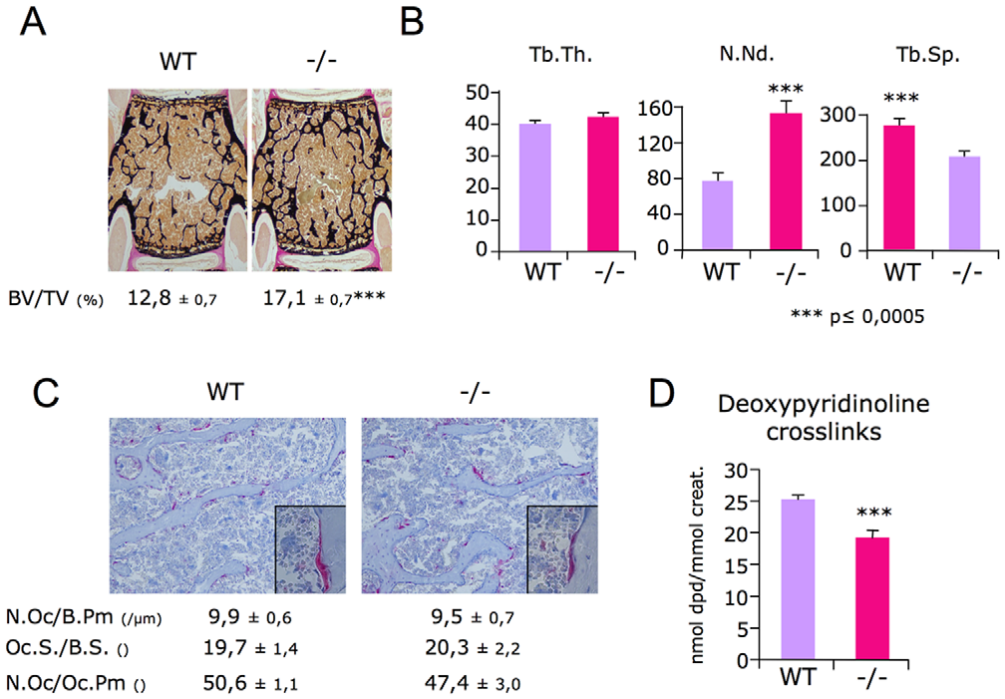
High bone mass and functional bone resorption defect in 3-month-old *Sema4D*-deficient females. A/ Bone volume and bone micro architecture parameters of 3-month-old WT and *Sema4D*-deficient mice were measured on vertebrae sections stained with von Kossa/Van Gieson stain. B/ B.V./T.V., bone volume/tissue volume; Tb.Th., trabecular thickness; N.Nd., number of nodes, Tb.Sp.; trabecular spacing n≥10 for both genotype. C/ *In vivo* quantification of osteoclast numbers and sizes of 3-month-old female vertebral sections stained for TRAP activity and with alcian blue, revealed that osteoclast differentiation is not affected in the absence of *Sema4D*. (N.Oc./B.Pm., number of Oc per bone perimeter; Oc.S./B.S., Oc surface per bone surface; N.Oc./Oc.Pm number of Oc per Oc perimeter) n = 8 for both genotypes. D/ Urinary deoxypyridinoline (DPD) cross-links elimination was significantly lower in *Sema4D* −/− animals compared to WT, indicating that the *Sema4D* deficiency results in a functional decrease in bone resorption n = 8 for WT and n = 14 for *Sema4D−/−* mice. Error bar represents SEM; *** indicate a p value≤0.0001 between two groups.

To determine if *Sema4D* has a function in bone, we first analyzed *Sema4D*-deficient mice using histomorphometry. In 3-month-old female vertebrae, quantification of the trabecular bone volume revealed a 33% increased bone mass in *Sema4D*-deficient mice compared to WT (Fig. 3A). Bone structure was also modified, the thickness of the trabeculae being unaffected, while the number of nodes and the trabecular spacing were significantly lower in *Sema4D*-deficient mice compared to WT, indicating that the number, but not the size, of the trabeculae was modified in the *Sema4D*-deficient mice (Fig. 3B). We then tested resorption parameters *in vivo*. Deoxypiridinoline crosslinks (DPD) were decreased in the *Sema4D*-deficient mice when compared to their WT littermates, showing that the increased bone mass resulted, at least in part, from decreased bone resorption (Fig. 3D). Neither the number nor the size of osteoclasts had been affected in the *Sema4D*-deficient mice, showing that the decreased bone resorption observed in *Sema4D*-deficient mice was due to a functional defect in the osteoclasts (Fig. 3C). On the other hand, the measured cellular and dynamic bone formation parameters were similar in *Sema4D*-deficient and WT mice (Fig. S1A–B). Altogether these data show that *Sema4D* is indeed a physiological regulator of bone resorption, without affecting bone formation.

### The bone resorption phenotype of *Sema4D*-deficient mice is gender dependent and is not osteoclast autonomous

**Figure 4:**
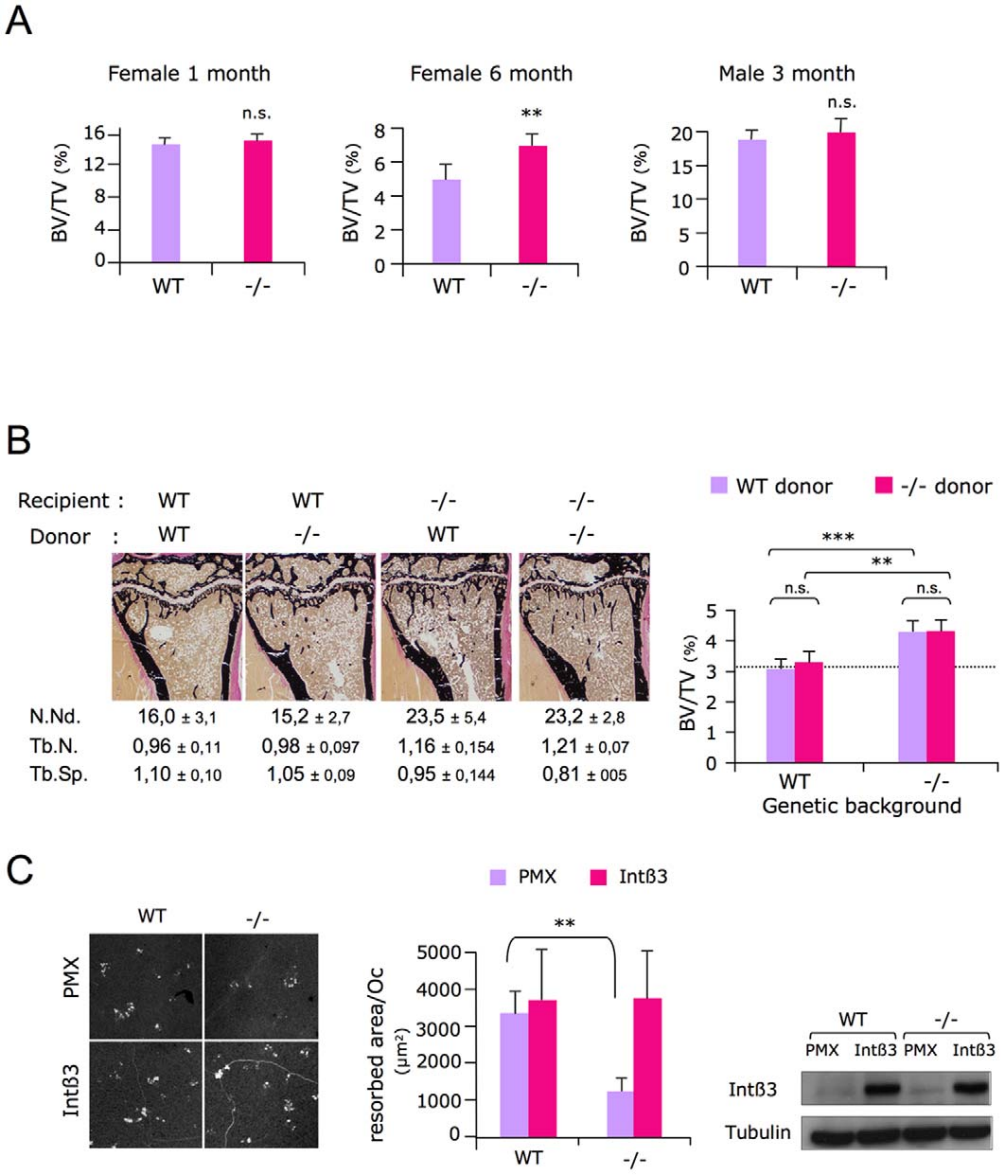
*In vivo* increased bone mass in *Sema4D* −/− mice is not due to an osteoclast defect. A/ Bone volume measurement of 1-month-old female and of 3-month-old male vertebrae by histomorphometrical analysis (respectively: n = 9 and n = 11 for WT; n = 9 and n = 12 for *Sema4D−/−* mice), and of 6-month-old femurs by microCT (n = 12 for WT and n = 9 for *Sema4D−/−* mice), revealed that the high bone mass phenotype of *Sema4D* −/− mice is only present in sexually mature females, but not in males. B/ 2-month-old *Sema4D* −/− and WT littermates received lethal doses of γ radiation and were subsequently transplanted with *Sema4D* −/− or WT bone marrow. The bone volume of female femurs was measured by histomorphometry 1 month after transplantation (i.e. in 3-month-old mice) n = 12 per group. C/ Defective resorption of *Sema4D*−/− osteoclasts is compensated by integrin ß3 chain overexpression. Micrographs of a representative experiment show the mineralized resorbed surfaces by transduced osteoclasts. The histogram shows that ß3 integrin chain over-expression in *Sema4D* −/− osteoclasts could rescue their resorption defects. Results represent the mean of two independent experiments (four ACC coverslips were used for each conditions). Total protein lysates of infected osteoclast progenitors were analyzed by western blotting for their ß3 integrin sub-unit content. It shows hß3 integrin expression in lanes Int ß3 compared to lanes corresponding to empty vector PMX transduced cell extracts. Equal loading was assessed by tubulin.

We further analyzed the bone phenotypes of WT and *Sema4D*-deficient females at earlier and later stages (1 and 6 months). These analyses revealed that the bone resorption phenotype was only present in sexually mature females, since 6-month-old females had increased bone mass similar to the 3-month-old mice, while 1-month-old *Sema4D*-deficient females had normal bone mass (Fig. 4A). On the other hand, 3-month-old *Sema4D*-deficient males had similar bone mass than their WT littermates (Fig. 4A). Those observations questioned the assumption that the bone phenotype observed in *Sema4D* −/− animals relied on an osteoclast-related intrinsic defect. To answer this question, we performed *Sema4D* −/− or WT bone marrow cross transplants in WT and *Sema4D*-deficient mice, following lethal doses of gamma radiation leading to hematopoietic lineage depletion, including that of osteoclasts. The results of these bone marrow cross transplants revealed that the bone phenotype of the transplanted mice did not depend on the donor genotype but on genetic background of the transplanted mice. Indeed, *Sema4D*-deficient mice had a higher BV/TV than the WT mice, whether they have been grafted with *Sema4D*-deficient or WT osteoclasts (Fig. 4B). Similarly, urinary DPD levels were higher in *Sema4D* −/− recipient mice than in the WT mice regardless of the graft genotype (Fig. S2). Thus, this unexpected result showed that the *Sema4D* −/− bone resorption phenotype is not secondary to an osteoclast-specific defect. In other words, the functional defects of *Sema4D* −/− osteoclasts that we observed *in vitro* are either too mild to be detected *in vivo* or are compensated for *in vivo*. Defective activities of osteoclasts lacking *Sema4D* have revealed an altered ß3 integrin sub-unit down-stream signaling, suggesting a common pathway for both proteins and a functional cross talk. To test this latter hypothesis, we transduced BMMs derived either from WT or *Sema4D* −/− mice, with empty retroviruses (PMX) or retroviruses expressing the full-length ß3 cDNA. After antibiotic selection, mature osteoclasts were differentiated and their ability to resorb mineralized matrix assessed. Over expression of the integrin ß3 chain in *Sema4D* −/− osteoclasts rescued their deficiencies. Indeed, integrin ß3 transduced *Sema4D* −/− osteoclasts were able to resorb mineral matrix to the same extent as WT osteoclasts (Fig. 4C). These results show that the integrin ß3 chain over expression can compensate for *Sema4D* −/− osteoclast resorption defects at least *in vitro* and indicate that *Sema4D* regulates bone resorption by an indirect mechanism *in vivo*.

### Reproductive defect in *Sema4D*-deficient mice

**Figure 5:**
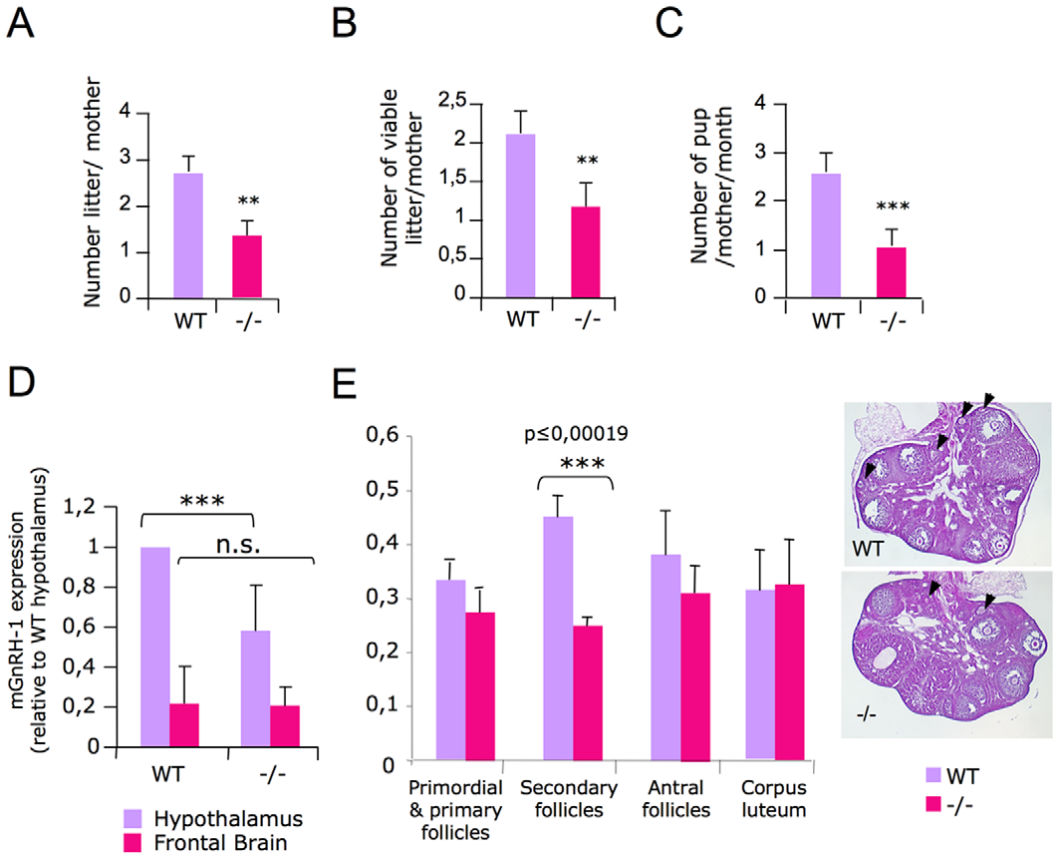
*Sema4D* deficiency decreases fertility and modifies the hypothalamic-pituitary-ovarian axis. A–C/ Monitoring of mating revealed a statistically significant decrease, in the number of litters per mother, of viable litters and the average number of pups per mother (number of cages n = 7 for WT and 11 for *Sema4D* −/−; performed twice). D/ *Gnrh1* expression in the *Sema4D* −/− adult female brain. Total mRNA was extracted either from total brain or hypothalamus of 3 month-old WT and mutant females. *Sema4D −/− Gnrh1* transcript levels were monitored by real time PCR and expressed as fold difference to the WT level, n = 4. E/ Morphological classification and quantification of follicles based on granulosa cell layers. Arrows indicate primordial or primary follicles containing an oocyte surrounded by a layer of granulosa cells. Graph shows the average number of follicles over 9 sections of each phenotype (n = 8).

While characterizing the origin of the *Sema4D −/−* mice high bone mass phenotype, we noticed that these mice produced fewer pups than WT, suggesting that *Sema4D* −/− mice were less fertile. Quantification of both the number of pups per brood and the number of litters per breeding period confirmed the decreased reproductive ability of *Sema4D*-deficient mice (Fig. 5A–D). We then asked how the *Sema4D* deficiency influenced the decreased fertility observed in these mice. It has been reported that deletion of *Sema4D* receptor plexinB1 (PlexB1) leads to a reduced number of *Gnrh1* secreting neurons in adult mice hypothalamus. We speculated though that *Sema4D*-deficient mice might present a similar phenotype [12]. Indeed, quantitative PCR revealed a 40% reduction in the *Gnrh1* transcript level in the hypothalamus of *Sema4D* −/− mice, whereas no variation was observed in frontal brain used as a tissue control (Fig. 5D). As gonadotropin-hormone releasing hormone-1 (*Gnrh1*) is involved in the hypothalamic-pituitary-ovarian axis, we then wondered if the decreased expression of *Gnrh1* could result in impaired ovarian function, which could explain the decreased fertility observed in *Sema4D −/−* mice. We compared the morphological follicles organization and counted the number of classified follicles in the ovaries of mutant versus WT mice. Analysis of histological ovary sections showed a significant reduced number of secondary follicles in the *Sema4D* −/− mice (Fig. 5E). On the other hand, ovary and uterus weights were similar in three-month-old *Sema4D* −/− females, compared to their WT counterparts (Fig. S3A–B). Accordingly, we did not find any significant variations in the basal levels of estradiol (E2) (Fig. S3B). Altogether our results show that *Sema4D* deficiency is required for proper female reproductive function.

### *Sema4D* regulation of bone resorption is dependent on female reproductive function

**Figure 6:**
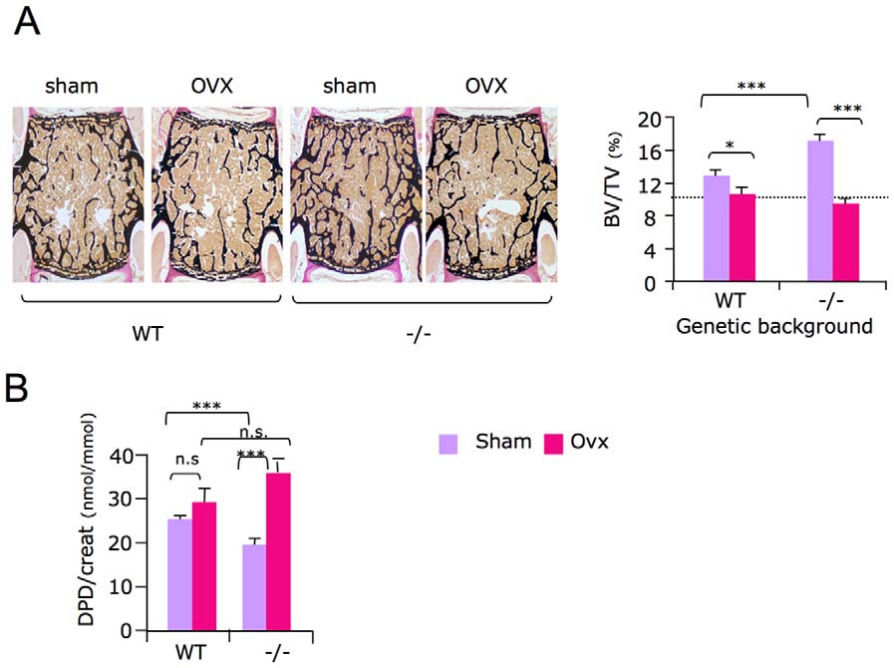
Increased bone mass in *Sema4D* −/− mice depends on ovarian function. A/ Bone volume quantification of 3-month-old *Sema4D* −/− and WT mice vertebrae one month after ovariectomy or sham surgery. B/ Quantification of urinary deoxypyridinoline residues from 3-month-old *Sema4D* −/− and WT mice, one month after ovariectomy or sham surgery show greater increases in bone resorption in *Sema4D* −/− than in WT, (n = 8). Error bar represents SEM; * p≤0.01; ** p≤0.001; *** p≤0.0001.

The bone marrow transplant experiment demonstrated that the bone resorption defect observed in *Sema4D* −/− mice did not result in an intrinsic osteoclast defect (Fig. 4B). Moreover this phenotype was observed only in sexually mature *Sema4D* −/− females, but not in males and the *Sema4D −/−* reproductive function was impaired (Fig. 4A and 5A–C). Altogether these results strongly suggested that the *Sema4D* bone resorption defect was dependent on ovarian function. To confirm this assertion, we ovariectomized 2-month-old *Sema4D*-deficient females and analyzed their bone phenotype one month later. As expected, WT ovariectomized mice had a lower uterine weight and lost about 20% of their tibial trabecular bone mass as a result of an increased bone resorption (Fig. 6A–B and S2A). On the other hand, following ovariectomy, *Sema4D*-deficient mice showed a 45% increase in bone resorption than WT mice (Fig. 6B). As a result, *Sema4D* −/− ovariectomized females had similar bone volumes to those of the WT ovariectomized females (Fig. 6A). The observation that the phenotype is osteoclast independent but sex dependent, together with impaired reproductive function, strongly suggests that the *Sema4D* −/− resorption phenotype is directly linked to ovarian function.

## Discussion

Although semaphorins were first identified as axon guidance cues, they have since, been shown to be important regulators of a wide variety of biological functions. The present study has uncovered new functions for semaphorin 4D as a regulator of bone resorption both *in vitro* and *in vivo*.

*In vitro*, *Sema4D* −/− osteoclasts are unable to resorb mineralized matrix properly and this defect is, at least in part, due to altered adhesion ability and subsequently to a migration defect. Remarkably these features are similar to those observed in *integrin ß3* −/− osteoclasts, such as defective adhesion, spreading and bone resorption [16]. Together with those similarities, we found that *Sema4D* deficiency in osteoclasts leads to sustain phosphorylation of components of the adhesion complex (Pyk2 and c-Src) (Fig. 2E). This also affects the activity of the downstream effector Rac-1 (Fig. 2F). Moreover, *Sema4D* −/− defective resorption is rescued by integrin ß3 chain over-expression.Thus our data show for the first time that *Sema4D* can act as a modulator of αVß3 integrin downstream signaling in osteoclasts and suggest functional cross-talk between *ß3 integrin* and *Sema4D* (Fig. 4C). This might be a general property of *Sema4D*. Notably, *Sema4D* and *ß3 integrin* are co-expressed in platelets and it is conceivable that the functional crosstalk between *ß3 integrin* and *Sema4D* also occurs platelets [9]. Strengthening this idea a very recent study reported that such a crosstalk between *Sema4D* and α*_IIB_ ß3* integrin takes place for thrombus formation by platelets [21].

In apparent agreement with the *in vitro* data showing functional defects in osteoclasts, we report that *Sema4D*-deficient females present a high bone mass phenotype linked to defective bone resorption. Histomorphometrical data show a functional rather than a differentiation defect of the osteoclasts in *Sema4D*-deficient females, since their osteoclast numbers and sizes did not differ from WT (Fig. 3B–C). However, this phenotype was gender specific, and only observed in sexually mature females. In addition, bone marrow cross transplant experiments confirmed that the decreased bone resorption *in vivo* was not due to an intrinsic defect in the osteoclasts (Fig. 4A–B). The discrepancy between our *in vitro* and *in vivo* data indicates that the functional defect of *Sema4D* −/− osteoclasts might be compensated for *in vivo* and that, unexpectedly, *Sema4D* controls bone mass mostly through an indirect pathway. Two non-exclusive mechanisms could explain these differences. First, our data suggest a functional cross-talk between ß3 integrin and *Sema4D* as it modulates ß3 integrin intracellular signaling, although they do not interact directly (data not shown). It is thus conceivable, that the *Sema4D* osteoclast-specific effect could be compensated, at least partially, *in vivo*, by ß3 integrin sub-unit. Second, *in vivo Sema4D* −/− high bone mass phenotype is dependent on mice hormonal status that dominates over the osteoclast intrinsic resorption defect. Therefore, the bone resorption defect observed in *Sema4D*-deficient mice is the result of an indirect mechanism.

In addition to the bone phenotype, we observed that reproduction is impaired in *Sema4D*-deficient mice, revealing a new physiological function of *Sema4D*. Our data showed that the *Sema4D*−/− defective gonadal function could, at least in part, be explained by a significant decrease in *Gnrh1* hypothalamic expression and reduced ovarian follicle maturation (Fig. 5D–E). These latter results obtained from the analysis of *Sema4D*−/− mice abnormalities strengthen two recent reports showing that *Sema4D* is indeed involved in hypothalamic-hypophyseal-ovarian axis development. First, *ex vivo*, *Sema4D* is implicated in mouse ovary follicular development [14]. Second, analysis of *Sema4D receptor PlexinB1*-deficient mice phenotype revealed that *PlexinB1* depletion results in a reduction of *Gnrh1*secreting neuron in mice adult brain due to a defective *Sema4D* induced neuron migration [12].

On the other hand no change was detected in *Sema4D* −/− mice body or uterine weights, in accordance with the fact that these mice have similar E2 blood basal levels as WT mice (Fig. S3A–B). Although no hypogonadism was detected in *Sema4D* −/− mice, they presented a decreased fertility. This observation reminds of clinical cases in women, in which primary ovarian deficiencies were observed along with normal E2 blood levels [22], [23]. Although we measured normal basal E2 blood level found in *Sema4D* −/− mice, we cannot also exclude a fine deregulation of E2 cycles. Alternatively, it is also possible that other hormones from the ovarian axis might explain the reproductive function defects and the secondary bone phenotype. Indeed, contribution of other than E2 hypothalamic-hypophyseal-ovarian axis hormones to bone mass regulation has largely been established [1], [24], [25]. For instance, it has been demonstrated that FSH directly regulate bone mass independently of E2 by activating bone resorption and therefore it might be a potential factor involved in *Sema4D* −/− bone resorption phenotype [24]. In the same way, we did not completely rule out the possibility that *Gnrh1* could act directly on osteoclasts, to promote bone resorption, although it has been shown that *Gnrh1* have no effect on osteoclastogenesis [24] and that hypogonadism is currently associated with bone loss [26]. In addition to the FSH direct effect on bone, recent studies have reported that elevated FSH levels increase bone formation, independently of E2, and that this increased bone formation is correlated to the FSH induced ovarian secretion of inhibin and androgens [27]. There are accumulating evidences showing that gonadal factors, such as inhibins, would regulate bone remodeling either acting on bone formation if their mode of exposure is continuous or bone resorption if it secreted in a cycling mode [28]. This complex endocrine regulation of bone mass by the inhibins can also be observed in woman prior to menopause [29], [30]. Thus inhibins might also be good candidates to play a role in the indirect regulation of bone mass by *Sema4D*.

Nevertheless, we have shown the dependence of the *Sema4D* −/− mice bone resorption phenotype on ovarian functions. Beside the gender specificity of the bone resorption phenotype, we found that the ovariectomy of sexually mature *Sema4D* −/− females abrogates the bone phenotype. This result clearly demonstrates the absolute requirement of mature ovaries to observe the bone phenotype in *Sema4D*-deficient female mice (Fig. 6A–B). In summary, our present study demonstrates first that *in vitro* the defective resorption activity of sema4D−/− osteoclast is due to dysfunction of adhesion and migration properties reflected by an hyperphosphorylation of ß3integrin sub-unit down-stream effectors. Secondly, we provide evidence that the observed high-bone mass phenotype of *Sema4D*−/− female mice is due to a functional defective resorption. We show that this *in vivo* effect of *Sema4D* on bone resorption does not result from an osteoclast-specific defect but is rather dependent on the ovarian function by an as-yet-undefined pathway. Thus *Sema4D* is an indirect regulator of bone resorption that acts through its effect on the reproductive system in females. In conclusion, *Sema4D* is a factor involved in the cross talk between bone remodeling and gonadal function.

## Materials and Methods

### Kits and Reagents

LSM (Lymphocyte Separation Media) and RNA-Now were purchased from Eurobio. α-MEM and fetal calf serum were from Invitrogen and Biowest, respectively. Ascorbic acid, sodium b-glycerophosphate, culture cell BSA and Leukocyte acid phosphatase kit for TRAP staining were from SIGMA. Vitronectin was purchased from BD Biosciences. RNeasy Kit, Quantitec primer assay and Mix-Quantitect SYBR Green were from Qiagen. I-ScripTM cDNA synthesis Kit was from BioRad. Antibodies: anti-*Sema4D* antibodies were BMA12, Abm30 clones and CD16/32 (clone2.4G2) respectively from Cliniscience and BD Biosciences. Anti-P-Pyk2 was from Biosource, anti-P-cSrc (Y-416) from Cell Signaling. Anti-cSrc, , goat anti ß3 integrin, was purchased from Santa-Cruz and hamster anti ß3 integrin from BD pharmingen. Secondary antibodies, mouse and rat IgGs were from Jackson.

The RNeasy Kit, Quantitec primer assay (QT 01062600) and Mix-Quantitect SYBR Green were from Qiagen. I-ScripTM cDNA synthesis Kit was from BioRad. Pyrilinks-D immunoassay and creatinin kits were used according to the manufacturer Quidel Corporation's protocols. E2 levels we measured by EIA from 2 consecutive days collected blood samples (Cayman, Chemical). Statistical differences between groups were assessed using *t*-tests.

### Mice

*Sema4D*-deficient mice were described in [18], were backcrossed with C57BL/6 mice. Background strain characterization by Charles Rivers genetic testing services indicated that 89,43% of the 110 tested markers were specific of the C57Bl6 background.

All mice were maintained in our animal facility and cared for in accordance with institutional guidelines for animal welfare (Approval was given by “the comité regional d'éthique pour l'expérimentation animale” on May 06 2008) with the register approval number 0265). For the induced sex hormone deficiency, 8-week-old female mice underwent ovariectomies (ovx), whereas the controls consisted of sham-operated mice (sham).

Statistical differences between groups were assessed using *t*-tests.

### Primary osteoblast cultures from calvaria

Osteoblasts were isolated from enzymatically dissociated calvaria from 3-day-old mice and were plated in differentiation medium: a-MEM) containing 10% heat-inactivated serum 50 g/ml ascorbic acid and 10 mM sodium ß-glycerophosphate. After 24 h, the cells were harvested using 0.01% trypsin in PBS and the cells were plated at 20 cells/mm^2^ and grown for 22 days in the same differentiation medium (replaced every two days).

### Primary osteoclast cultures

Bone marrow mononuclear cells from 8-week-old WT or *Sema4D* −/− mice were cultured at 500cells/mm^2^ for 5 to 6 days in the presence of a-MEM containing 10% (v/v) fetal calf serum, M-CSF (20 ng/ml) and GST-RANKL (30 ng/ml) [31]. We designated as pre-osteoclasts, cells containing 2 to 3 nuclei, obtained after 3 days of culture, whereas osteoclasts were multinucleated cells showing 5 to 10 nuclei and mature osteoclasts were large multinucleated cells with a high number of nuclei (n≥10). TRAP activity in cells was assessed after staining with leukocyte acid phosphatase kit (Sigma).

### Quantification of proliferation

At each time point cells seeded on multi-well plates are fixed in 1,1% glutaraldhedyde for 15 min and washed twice in water and air dried. Prior lysis in acetic acid 10%, cells are stained in crystal violet 0,1% for 20 min, washed and air dried, plates are read at OD 590 nm for quantification.

### Adhesion and spreading assay

For *in vitro* spreading assays, osteoclasts were detached with 0.02% EDTA in PBS and washed twice with a-MEM containing 10 mM Hepes and 0.1% culture cell BSA. They were kept in suspension for 2 hours at 37°C and then either maintained in suspension or plated on 5 µg/ml vitronectin-coated dishes, according to the manufacturer's instructions. At the stated time, cells were lysed, and an equal amount of protein was analyzed by Western blotting.

For the spreading assay, osteoclasts were seeded on 5 µg/ml vitronectin pre-coated cover slips for 5, 10, 15 or 30 min and fixed with 4% PFA, permeabilized and F-actin labeled with 488-Alexa-Fluor phalloidin. Cells were imaged with a planNeofluar 20× objective (NA 0.5) using a Zeiss axioplan microscope. The surface area was automatically measured with ImageJ software, using an ImageJ plug-in developed by our imaging facility (Platim IFR128).

### BMMs retroviral infection

PMX derived retroviral particles were produced in Plat-E retroviral packaging cells (Cell Biolabs). In parallel PMX -IRES-GFP retrovirus (from Dr. T. Kitamura –Tokyo) was produced using the same conditions to be able to visualize and follow transfection and infection under an inverted microscope. Briefly, on day 1, PMX DNA constructs (PMX empty, or expressing human integrin ß3 chain) were transfected in Plate-E cells with Fugen 6 (Roche), on day 2 fresh media was added. The media was collected on day 4 and centrifuged to remove the cell debris. M-CSF (20 ng/ml) and 8 µg/ml of polybrene (Sigma) were added to supernatant, which was used to infect overnight bone marrow mononuclear cells prepared on day 3 in presence of MEM 10% FCS and 25 ng/ml of M-CSF. The following day, the inoculum was removed and the transduced cells were subjected to blasticidin selection (1 µg/ml) for 2 days in presence of MEM 10% FCS and 20 ng/ml of M-CSF. Then medium was replaced by classical osteoclast differentiation medium (MEM 10% FCS 20 ng/ml of M-CSF and 30 ng/ml of RANK-L for an additional 3days before lifting and seeding the mature osteoclasts on mineral for 24 h [32].

### Cell transfer experiments

Two-month-old wild-type or *Sema4D* −/− mice were irradiated with lethal doses of gamma radiation (10 Gray) and reconstituted with a retro-orbital injection of 1 million bone marrow cells derived from wild-type or *Sema4D* −/− mice, un-transplanted mice died after 10 days. One month after grafting, the mice were sacrificed for bone analysis.

### Histology and histomorphometric analyses

Non-decalcified bones were embedded in methyl methacrylate, before being stained for Von Kossa, TRAP staining or histological analysis. Mice were injected with 100 µl of a 5 mg/ml of a calcein solution, 6 and 2 days before sacrifice and were processed for plastic embedding, as previously described [33]. Measurements were performed with the OsteoMeasure Analysis System (Osteometrics) using a 3CCD color video DXC-390camera (Sony) coupled to a Leica microscope, according to standard procedure [34].

### Morphological classification of follicles

Three-month-old females were sacrificed. Frozen ovaries were sectioned and hematoxylin stained. Results were obtained through averaging all serial sections (200 µM). Follicles were classified according to Myers [35].

### RNA extraction and RT-PCR

Real-time RT-PCR was performed using the MXP-3000P PCR-system (Stratagene). Samples were run in triplicate and the cycle parameters were 95°C for 15 min, followed by 40 cycles at 95°C for 30 s, 55°C for 30 s and 72°C for 30 s. Gene expression levels were calculated using the ΔΔCt method. Expression was normalized using the mouse ribosomal gene (Rps17).

### Statistical analysis

Statistical differences were analyzed with Student's t-test and error bars represent SEM. Values of *P*≤0,01 were considered significant. * p≤0.01; ** p≤0.001; *** p≤0.0001 when not otherwise specified.

## Supporting Information

Figure S1 A/ Cellular and B/ Dynamic bone formation parameter measurements using alcian blue and double calcein labeling, respectively, show an absence of bone formation defects in 3-month-old *Sema4D* −/− females compared to their WT littermates. (N.Ob./B.Pm., number of Ob per bone perimeter; Ob.S./B.S. Ob surface per bone surface; BFR, bone formation rate; MAR, mineral apposition rate; M.S./B.S., mineralized surface per bone surface). Error bar represents SEM, *** indicate a p value≤0.001 between two groups (n = 12).
(TIF)

Figure S2 Urinary DPD measurement of 3-month-old *Sema4D* −/− and WT irradiated mice one month after transplantation (n = 10 for WT and n = 12 for *Sema4D*−/− respectively).
(TIF)

Figure S3 A/ Uterine weight of 3-month-old *Sema4D* −/− and WT mice: after ovariectomy or sham surgery (n = 7 and 8 respectively). B–C/ No significant differences were observed in ovary weight and estradiol levels in 3 month-old females (n = 8 and n = 20 respectively). Error bar represents SEM, *** p≤0.005.
(TIF)
