# Supplementary figures and images for: Control of Bone Resorption by Semaphorin 4D Is Dependent on Ovarian Function
Source: PLoS One. 2011 Oct 26;6(10):e26627. doi: 10.1371/journal.pone.0026627 (PMC3202567; doi:10.1371/journal.pone.0026627)

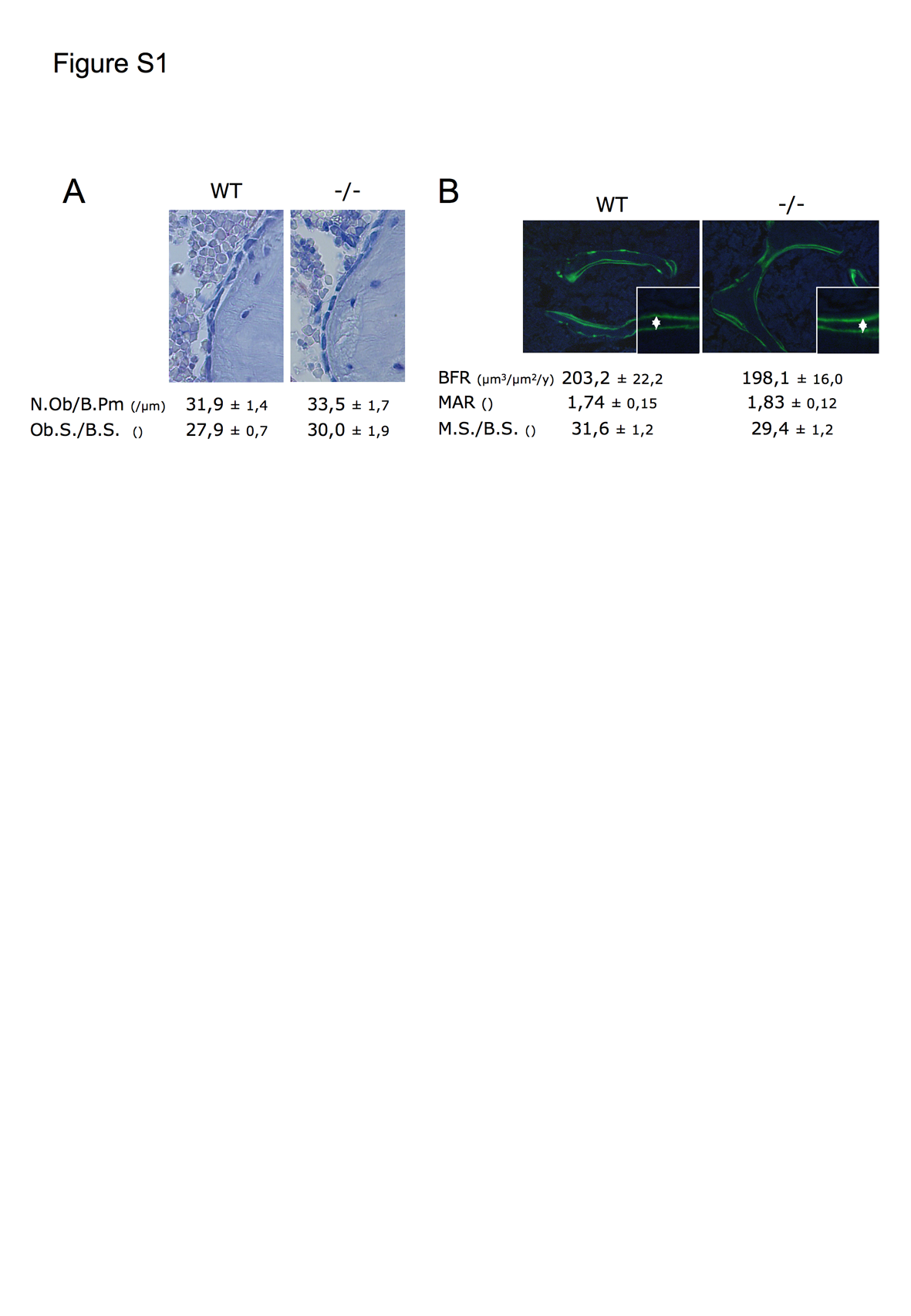

Supplement: Figure S1 — A/ Cellular and B/ Dynamic bone formation parameter measurements using alcian blue and double calcein labeling, respectively, show an absence of bone formation defects in 3-month-old Sema4D −/− females compared to their WT littermates. (N.Ob./B.Pm., number of Ob per bone perimeter; Ob.S./B.S. Ob surface per bone surface; BFR, bone formation rate; MAR, mineral apposition rate; M.S./B.S., mineralized surface per bone surface). Error bar represents SEM, *** indicate a p value≤0.001 between two groups (n = 12). (TIF) [file pone.0026627.s001.tif]

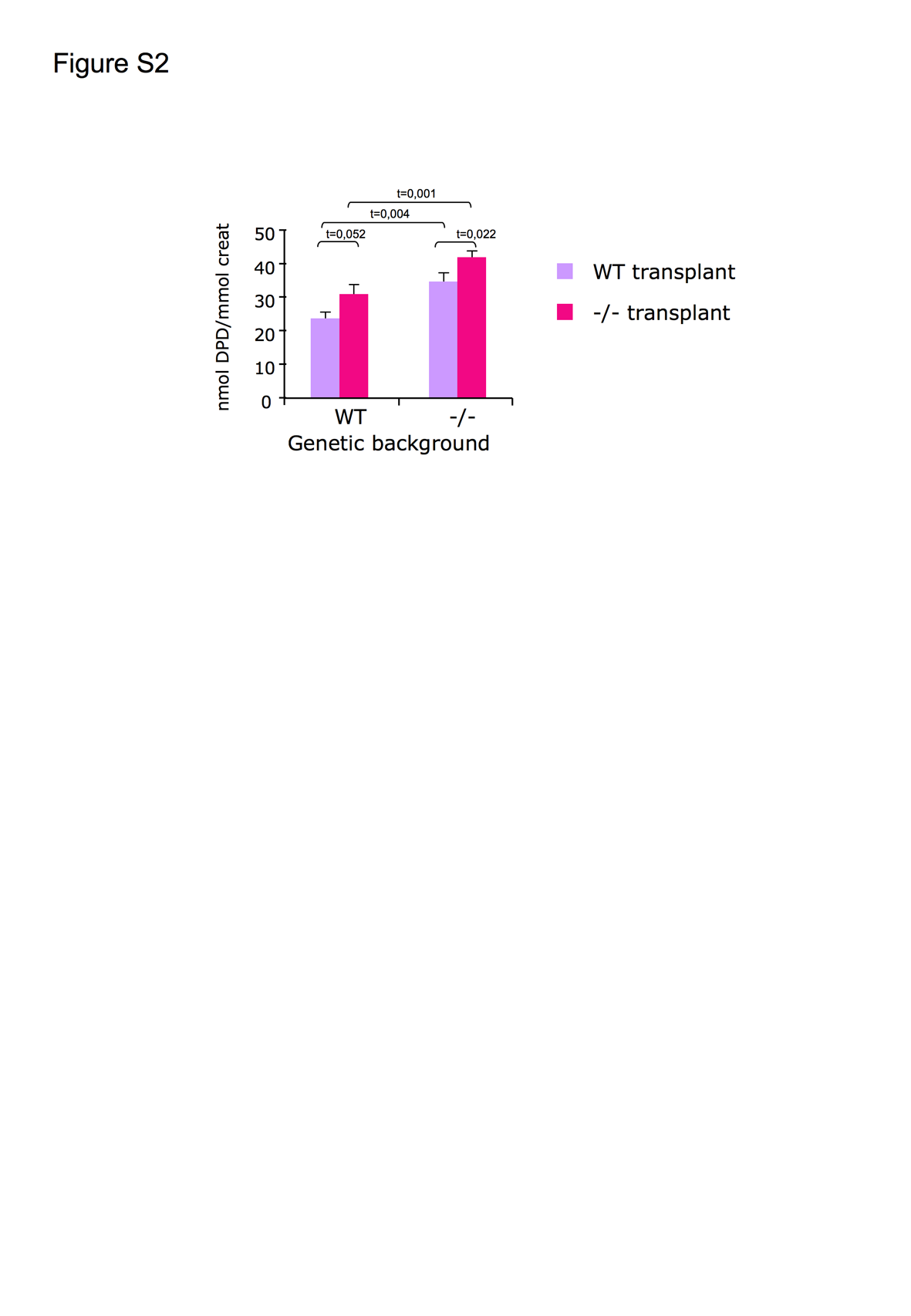

Supplement: Figure S2 — Urinary DPD measurement of 3-month-old Sema4D −/− and WT irradiated mice one month after transplantation (n = 10 for WT and n = 12 for Sema4D−/− respectively). (TIF) [file pone.0026627.s002.tif]

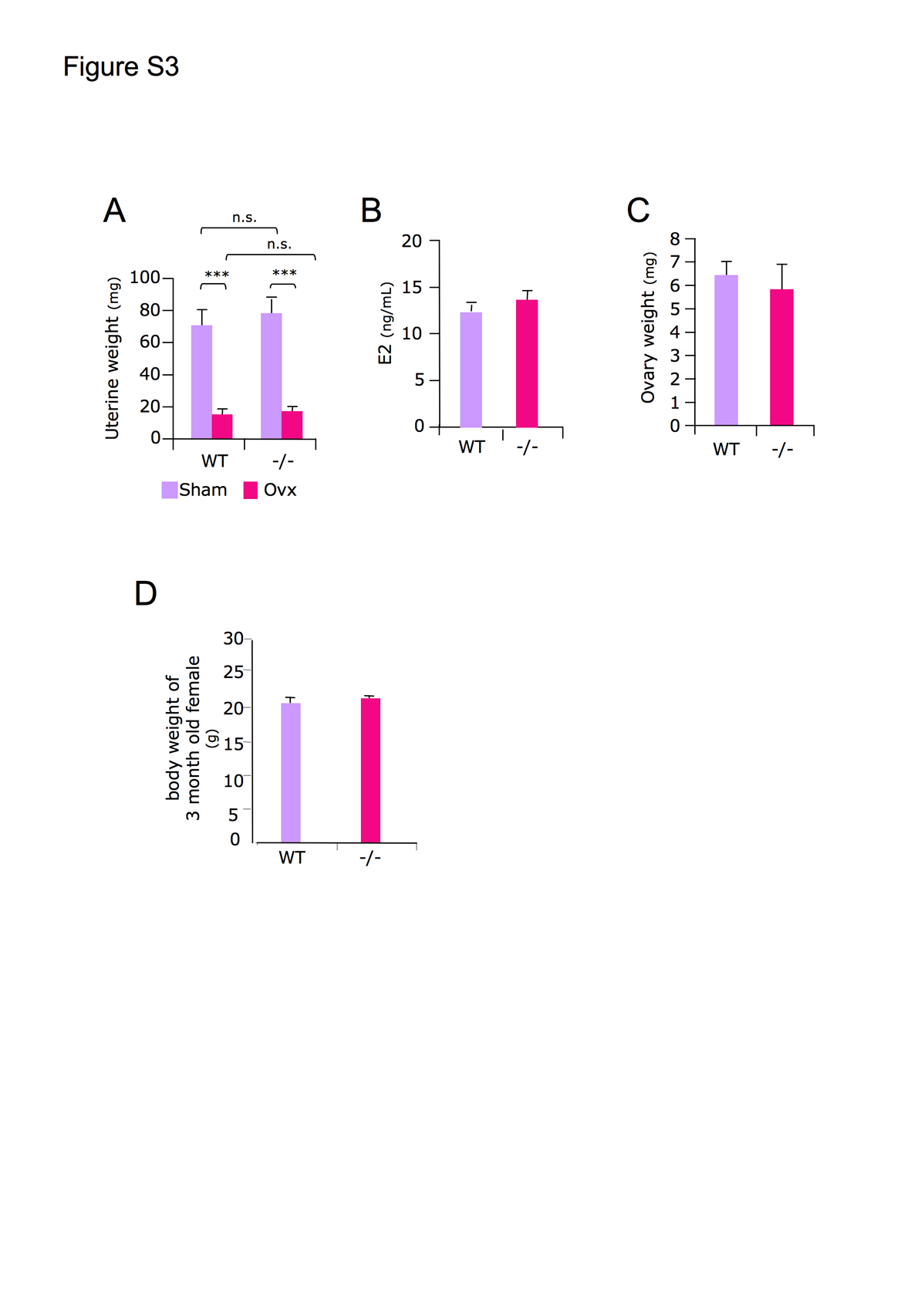

Supplement: Figure S3 — A/ Uterine weight of 3-month-old Sema4D −/− and WT mice: after ovariectomy or sham surgery (n = 7 and 8 respectively). B–C/ No significant differences were observed in ovary weight and estradiol levels in 3 month-old females (n = 8 and n = 20 respectively). Error bar represents SEM, *** p≤0.005. (TIF) [file pone.0026627.s003.tif]
